# A Review on Pulsed Laser Fabrication of Nanomaterials in Liquids for (Photo)catalytic Degradation of Organic Pollutants in the Water System

**DOI:** 10.3390/nano13192628

**Published:** 2023-09-23

**Authors:** Yang Li, Liangfen Xiao, Zhong Zheng, Jiujiang Yan, Liang Sun, Zhijie Huang, Xiangyou Li

**Affiliations:** 1 College of Electrical Engineering, Naval University of Engineering, Wuhan 430033, China; 2 Wuhan National Laboratory for Optoelectronics (WNLO), Huazhong University of Science and Technology, Wuhan 430074, China; 3 Department of Basic Courses, Naval University of Engineering, Wuhan 430033, China

**Keywords:** nanomaterials, pulsed laser heating in liquids, organic pollutants, catalytic removal, massive production

## Abstract

The water pollution caused by the release of organic pollutants has attracted remarkable attention, and solutions for wastewater treatment are being developed. In particular, the photocatalytic removal of organic pollutants in water systems is a promising strategy to realize the self-cleaning of ecosystems under solar light irradiation. However, at present the semiconductor-based nanocatalysts can barely satisfy the industrial requirements because their wide bandgaps restrict the effective absorption of solar light, which needs an energy band modification to boost the visible light harvesting via surface engineering. As an innovative approach, pulsed laser heating in liquids has been utilized to fabricate the nanomaterials in catalysis; it demonstrates multi-controllable features, such as size, morphology, crystal structure, and even optical or electrical properties, with which photocatalytic performances can be precisely optimized. In this review, focusing on the powerful heating effect of pulsed laser irradiation in liquids, the functional nanomaterials fabricated by laser technology and their applications in the catalytic degradation of various organic pollutants are summarized. This review not only highlights the innovative works of pulsed laser-prepared nanomaterials for organic pollutant removal in water systems, such as the photocatalytic degradation of organic dyes and the catalytic reduction of toxic nitrophenol and nitrobenzene, it also critically discusses the specific challenges and outlooks of this field, including the weakness of the produced yields and the relevant automatic strategies for massive production.

## 1. Introduction

As one of the most significant resources of life, the quality of water directly determines the safety of human beings and other creatures. However, with the development of industrial and agricultural production, large amounts of hazardous products, such as textile dyes [1], chemical fertilizers [2], antibiotics [3], petroleum [4], and pesticides [5], are poured into wastewater streams; these products have caused the organic pollution and stench of water, seriously threatening human health and the whole ecosystem. In particular, the release of textile dyes and pesticides has induced the metamorphism of water, and people who depend on this polluted water become ill and even die; meanwhile, the toxic products are also harmful to the creatures in the water due to the circulation of the food chain [1,5]. Hence, how to remove these toxic products and suitably enable the recovery of the water system is worthy of consideration. Since the pioneering work of semiconductor-based photocatalysts demonstrated by Fujishima and Honda in 1972 [6], the photocatalytic degradation of organic pollutants under solar light irradiation has been considered a promising approach to the realization of self-cleaning water systems. This is because a redox reaction for pollutant oxidation can be obtained between light-generated electron–hole pairs and water molecules via an advanced oxidation process (AOP) [7,8]. Unfortunately, for most of the semiconductor materials, such as TiO_2_, ZnO, SnO_2_, and CeO_2_ [9,10,11,12], their bandgaps are generally wide (exceeding 3.0 eV). They are only sensitive to ultraviolet light (λ < 400 nm), while a mere 4% of ultraviolet components are contained in solar light; so, the charge carriers can rarely be generated by solar light. Thus, the photocatalytic activity is significantly lower, which restricts the massive application of this technology [13,14].

In previous reports, to enhance the photocatalytic abilities of catalysts, some nanocomposites and nanostructures have been developed, including the metal–semiconductor nanocomposites [15], the metal–graphene nanostructures [16], and the carbon-based metal–nanocomposites [17]. From these cases, constructing a hybrid heterojunction by surface modification proved to be an effective way to enhance photocatalytic performances in hazardous material removal. In particular, the Z-type and S-type heterojunctions, which are mainly defined by the transferring routes of light-generated charge carriers, were proposed (the geometrical shapes of the transfer path were similar to the letters “Z” or “S”, respectively), and these heterojunctions were widely utilized to boost the visible light harvesting and separation of the excited charge carriers [15,16,17] because the synergistic effect of nanocomposites or nanostructures is beneficial for overcoming the drawbacks of a single semiconductor. For instance, as listed in Table 1, Liu and Shen et al., using the hydrothermal method, developed a ZrS_4_-Znln_2_S_4_ nanocomposite by anchoring ZrS_4_ on the surface of Znln_2_S_4_ nanosheets. The introduction of active ZrS_4_ promoted the formation of migrating Zr-S_4_ sites, which induced the interfacial modulation of Znln_2_S_4_ and the perturbation of the bandgap structure so that more reactive oxygen species (ROS) could be generated for the photocatalytic degradation of tetracycline, where there was a 3-fold enhancement compared with neat Znln_2_S_4_ [18]. Li and Liu et al. prepared a core–shell or hollow Ag/ZnO nanocomposite using a 532 nm pulsed laser that selectively melted Ag nanoparticles in a ZnO-containing colloidal suspension. The photocatalytic performance of prepared hybrid nanocomposites was enhanced two times compared with that of neat ZnO for the degradation of methylene blue dyes in a water system, with a higher rate constant of 0.04 min^−1^. This is because the coupling effect of the Ag component promoted the visible light absorption ability of the nanocomposite via a unique surface plasmon resonance (SPR) effect [19]. Zeng, Cai, Xiong, et al. fabricated a multilayer graphene/CoO@Co nanocomposite by using a facile impregnation–calcination method. The CoO@Co nanoparticles were embedded in the derived multilayer graphene. Combined with the potential of peroxymonosulfate (PMS), based on the powerful AOP effect in wastewater treatment, the photocatalytic activity for chlortetracycline hydrochloride removal was maximally optimized, and a 100% removal ratio could be obtained within only 12 min [20]. 

In recent years, Drmosh et al., using pulsed laser ablation in liquids, constructed a hybrid Z-scheme TiO_2_/BP/g-C_3_N_4_ and WO_3_/BP/g-C_3_N_4_ nanostructure, respectively [21,22]. For overall water splitting, the prepared TiO_2_(WO_3_)/BP/g-C_3_N_4_ displayed a stable and efficient photocatalytic performance under simulated sunlight irradiation (xenon lamp, AM 1.5G, 100 mW/cm^2^). After inserting the components of g-C_3_N_4_ and constructing a Z-type heterojunction on the surface of TiO_2_/BP substrates, their photocatalytic activities were enhanced 5.4-fold (TiO_2_/BP/g-C_3_N_4_) and 5.3-fold (WO_3_/BP/g-C_3_N_4_) compared with that of a bare TiO_2_/BP nanocomposite, respectively. Gholami and Ritala et al., using a chemical approach, prepared an oxygen and nitrogen plasma-modified graphene (NOG) to support a nitrogen-doped ZnCuCo layered double hydroxide (LDH) nanocomposite (NLDH-NOG) [23]. For the photocatalytic production of hydrogen and the degradation of sulfanilamide (SA) under the illumination of an LED light (40 W, 427 nm, Kessil), the activities of prepared nanocomposites were synchronously enhanced and were 4.5-fold and 1.9-fold greater than that of pure LDH-OG, respectively [23]. The same group also synthesized dodecylbenzenesulfonate (DBS) modified ZnCuCo layered doubled hydroxide microspheres (DBS-ZnCuCo LDH) through a facile templet-free hydrothermal method [24]. The products also displayed a higher regeneration rate (3700 μmol g^−1^h^−1^) than that of bare ZnCuCo (2400 μmol g^−1^h^−1^) for photocatalytic hydrogen production under the same light illumination as above [24]. Khataee et al. synthesized a three-dimensional nitro-doped magnetic WO_3−x_@mesoporous carbon nanocomposite (NM-WO_3−x_@MC) by evaporation-induced self-assembly using diatom frustules as a natural template [25]. For the photocatalytic generation of hydrogen under the illumination of an LED lamp (300 W, λ > 420 nm, Shenzhen StarVanq Tech., Shenzhen, China), a 2765 μmol g^−1^h^−1^ production rate can be achieved, which is about 5.2-fold higher than that of pure WO_3_ (532 μmol g^−1^h^−1^) [25]. In addition, Shandilya and Priye et al. also summarized a series of two-dimensional carbonaceous materials (Graphene, GO, GCN, GDY, MXenes) for photocatalytic water detoxification and energy conversion [26].

Among the construction methods introduced above, which are different from the conventional chemical methods (hydrothermal, impregnation–calcination, and self-assembly), there are special characteristics of pulsed laser heating in liquids. The whole process does not contain chemical reactions; so, the quality of laser-treated products is very neat [27,28]. The multi-precursor materials dispersed in the liquid medium can be selectively heated and composited because the band structures and melting/boiling points for nanomaterials are different. For example, the noble metal components (no bandgap structures or the bandgaps are extremely narrow) with higher laser absorption can be gradually heated, melted, and fragmented and subsequently loaded on the surface of semiconductors, while the semiconductors with lower laser absorption may be barely affected due to the restriction of their wide bandgaps (such as with TiO_2_, ZnO, SnO_2_ and CeO_2_; their bandgaps exceed 3.0 eV, and the light absorption is prevented) [29,30]. 

To date, several reviews have concentrated on pulsed laser fabrication of nanomaterials in liquids and their applications in catalysis, demonstrating the great potential of the pulsed laser fabrication of nanomaterials for energy conversion [31,32,33]. Specifically, Müller et al. gave an overview of pulsed laser-made nanomaterials for photo/electrocatalysis in water oxidation, oxygen reduction, and hydrogen evolution [31]. Choi et al. summarized the pulsed laser synthesis of metal nanoparticles, oxides, and carbon materials for various photo/electrocatalytic applications [32]. Koshizaki et al. presented a research overview of pulsed laser melting (PLM) in liquids for crystalline spherical particle preparation and its applications in photochemical/thermal treatments [33]. In this review, with regard to the relevant applications in environmental science, we mainly focus on the various pulsed laser-fabricated nanomaterials for the catalytic degradation of organic pollutants in water systems. Here, the smart intelligence of the pulsed laser preparation of nanomaterials and their high performances in environmental science are summarized. Moreover, this review not only highlights the innovative works in this field but also discusses the specific challenges and prospects in detail.

## 2. Pulsed Laser Fabrication of Nanomaterials in Liquids

For the photocatalytic degradation of organic pollutants, the development of a photocatalyst with a neat surface, high crystallinity, and low cost is extremely significant because the defects that exist in the photocatalyst will induce a high recombination rate of light-generated charge carriers [34,35]. As an innovative approach, pulsed laser irradiation in liquids demonstrates the characteristics of rapid heating and liquid quenching due to its short-pulsed widths (ns, ps, and fs magnitudes) and high repetition rates. Hence, there is a notable heat effect between the laser beam and nanomaterial interactions; consequently, the nanomaterials with high crystallinity can be rapidly prepared by pulsed laser heating in liquids, and their photocatalytic performances can be sufficiently displayed. In addition, the pristine nanomaterials after laser heating can be melted and can even explode in the liquid phase. Their plasma species are synchronously confined by the rapid quenching of the liquids; so, their crystalline structures can be naturally formed; in these structures, the light-generated charge carriers can be effectively separated without the prevention of massive defects [36].

**Figure 1:**
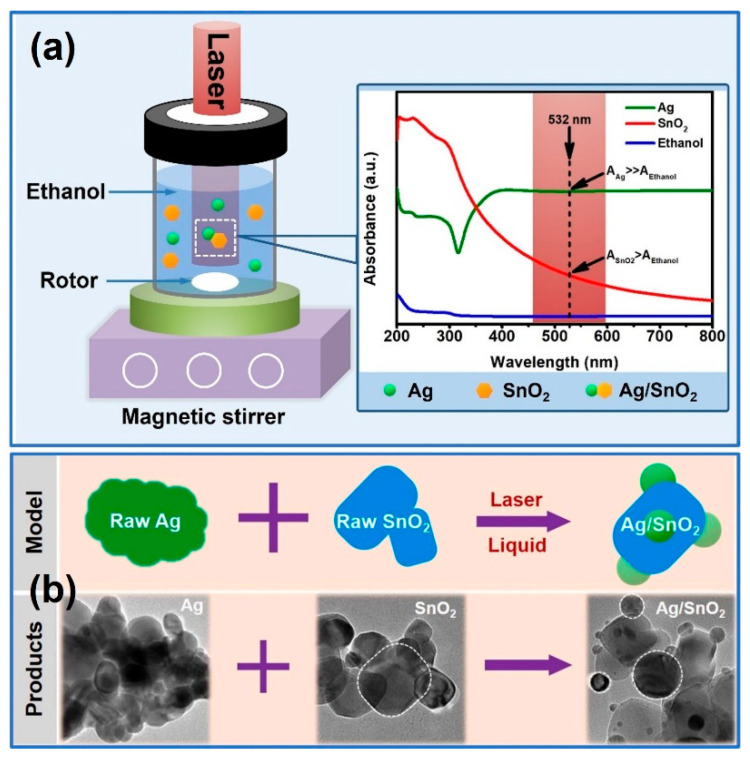
Schematic diagram (**a**) and morphological image (**b**) of Ag/SnO_2_ nanocomposite prepared by pulsed laser irradiation in liquid. Reprinted with permission from Ref. [37].

Another feature of pulsed laser irradiation in liquids is the selective heating effect on colloidal particles under a modest laser energy density. For example, as shown in Figure 1a, for pulsed laser unfocused irradiation to prepare Ag/SnO_2_ nanocomposites in ethanol solvent, the different optical absorptions between pure Ag, pure SnO_2_, and ethanol solvent are presented. Ag nanoparticles are much higher than SnO_2_ nanomaterials and ethanol solvent, which is under 532 nm, while the ethanol solvent is the lowest and is even transparent. Meanwhile, the melting point of Ag (about 962 °C) is much lower than that of SnO_2_ (about 1630 °C). Therefore, these intrinsic differences determine that Ag nanoparticles can be selectively heated by using a 532 nm laser beam once the heating effect is taking place, and the melting Ag components can be collided and composited with SnO_2_ nanomaterials under the uniform rotation of a small magnetic rotor [37]. Due to this selective heating effect on Ag nanoparticles, their average particle size, morphology, crystal structure, and even optical or electrical properties can be precisely controlled in the nanoscale under the tunability of laser parameters, including wavelength, energy density, pulsed width, and irradiation time. Meanwhile, during the synthesis process of nanocomposites, because the laser heating effect on different nanomaterials is a physical melting process, the single component of Ag can be controlled and loaded on the surface of SnO_2_ (the schematic diagram and morphological image are illustrated in Figure 1b); thus, the hybrid heterojunction between multiple components can be selectively constructed by pulsed laser-induced deposition in a liquid medium.

**Figure 2:**
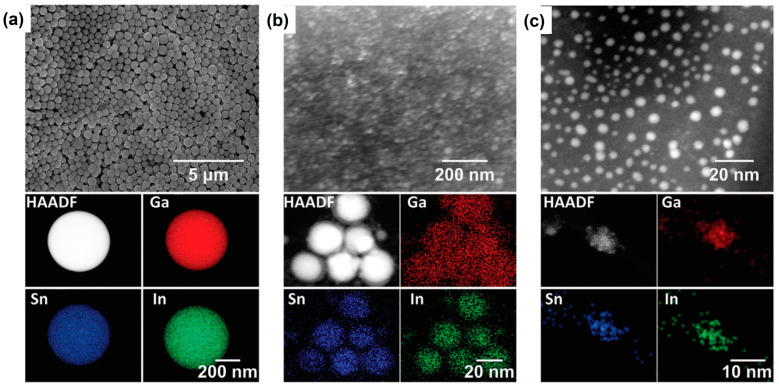
The SEM images and elemental mapping of Galinstan nanocomposites fabricated by pulsed laser irradiation under different laser fluences: (**a**) 75 mJ/pulse cm^2^, (**b**) 100 mJ/pulse cm^2^, (**c**) 175 mJ/pulse cm^2^, respectively. Reprinted with permission from Ref. [38].

In addition, the nanomaterials fabricated by using pulsed laser irradiation in liquid can be uniformly constructed under the rotation of a magnetic rotor or with ultrasonication technology. This precise modification of elemental components also provides an effective way to boost the photocatalytic or photo/electrochemical activities. For instance, Figure 2 illustrates a series of morphological images of laser-fabricated Galinstan nanocomposites (eutectic alloys of Ga, ln, and Sn) with different laser fluences (75, 100, and 175 mJ/pulse cm^2^, respectively) and their elemental mapping under the high-angle annular dark field (HAADF) [38]. The elemental distributions of Ga, Sn, and ln are uniformly displayed in the same area, confirming that the hybrid Galinstan nanocomposites are successfully pre-treated. During this laser processing, an ultrasonication rod was utilized to ensure a homogeneous reaction between the laser and the dispersed liquid metals. Taking the laser-fabricated Galinstan nanocomposites as the electron mediator in hybrid perovskite solar cells, the defects of the crystal lattice were modified by well-dispersed Ga, Sn, and ln components. Herein, Du, Wang, et al. obtained an excellent photoconversion efficiency (PCE) of 22.03% via this facile surface engineering [38].

The interactions between pulsed laser beams and nanomaterials are diverse, but the phase transition occurs gradually. On one hand, for single pure nanomaterials dispersed in the liquid medium, their fundamental properties (such as size and the relevant quantum effects) can be tuned by rapid laser heating under different laser conditions. The phase transition process can be theoretically described by using the classical “Heating-Melting-Evaporation” (HME) model [39,40]. On the other hand, for the multi-nanomaterials dispersed in the liquid medium, because of their intrinsic differences in optical and thermodynamical properties, the different components can be selectively heated by pulsed laser irradiation in a liquid medium; thus, the modification of surface components can be obtained to optimize their photocatalytic performances. Meanwhile, compared with the nanomaterials prepared by conventional chemical routes, the precise control of composition, particle size, crystallinity, crystallographic phases, defect densities, and the relevant catalytic properties is inherently difficult, because the chemical reactions cannot occur precisely under practical conditions, and surfactants are usually needed [31]. While the laser-fabricated nanomaterials do not contain any chemical reactions [31,33], the heating effect is a physical process. So, the surfaces of the prepared products are very neat, without the introduction of other chemical groups to prevent the fast migration of light-generated charge carriers, and the ROS can be produced more effectively to remove the organic pollutants in the water system.

## 3. Laser-Made Nanomaterials for (Photo)catalytic Degradation of Pollutants

### 3.1. Construction of Photocatalytic System for Pollutant Degradation

**Figure 3:**
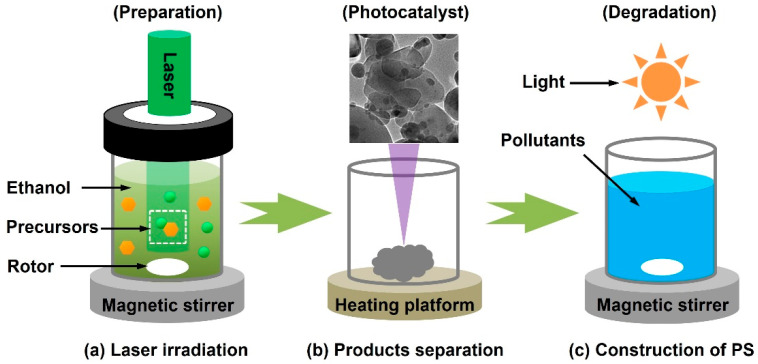
A schematic diagram for the construction of a photocatalytic system: (**a**) laser irradiation, (**b**) product separation, (**c**) construction of photocatalytic system.

In recent years, inspired by the smart intelligence of pulsed laser irradiation in a liquid medium, this technology has been utilized to fabricate various nanomaterials for the photocatalytic degradation of organic pollutants, such as organic dyes, antibiotics, and pesticides, in water systems [41,42,43]. In the experimental study, the photocatalytic system (PS) is usually constructed within three steps. As shown in Figure 3, the first step is the fabrication of functional nanomaterials under laser beam irradiation in liquids; the precursors can be selectively heated to form the colloidal suspension with a magnetic rotation. After that, the photocatalysts can be conveniently obtained by product separation via a heating platform to remove the liquids. This is because the photocatalytic process is the interaction between the catalyst and pollutant under light illumination; hence, the separated photocatalysts should be uniformly mixed with organic pollutants with the assistance of a magnetic stirrer to construct the PS.

### 3.2. Photocatalytic Degradation of Organic Dyes in the Water System

In general, for the photocatalytic degradation of organic dyes in water systems, the removal of dyes is usually attributed to the unique AOP technology [44,45]. During this process, the charge carriers from electron–hole pairs can be generated on the valance band (VB) and conduction band (CB) of semiconductor materials under the proper light illumination, respectively. The electrons distributed on the conduction band migrate to combine with the dissolved oxygen species in the water system to produce the superoxide radicals (the symbol is marked as: ⸳O_2_^−^). The process can be generally described as follows: semiconductor + proper light→e^−^ (CB) + h^+^ (VB), and e^−^ (CB) + O_2_ (H_2_O)→⸳O_2_^−^ [44]. Meanwhile, the relevant holes with powerful oxidation abilities can be reacted with the water species to produce the hydroxyl radicals (the symbol is marked as: ⸳OH), which can be simply described as h^+^ (VB) + H_2_O→⸳OH [45]. These reactive radicals with powerful oxidization abilities can be in contact with the organic dyes to destroy their molecular structures; thus, the diverse organic dyes can be removed by these redox reactions. The degraded organic products are finally transformed into CO_2_, H_2_O, and other chemical groups, and the relevant work is listed in Table 2.

**Figure 4:**
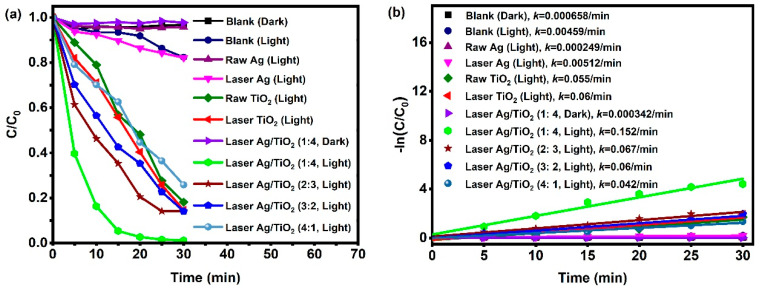
The photocatalytic activities of laser-prepared Ag/TiO_2_ nanocomposites. (**a**) The curve for MB degradation with the time evolution. (**b**) The corresponding dynamics fitting curves of MB concentration evolution. Reprinted with permission from Ref. [46].

The laser-prepared semiconductor materials provide a rapid approach to the narrowing of their bandgaps due to fact that their components can be precisely modified to boost the visible light harvesting by controlling the initial concentrations or mass ratios of the precursor materials. Hence, a series of desirable photocatalysts can be designed. These treatments and modifications are also beneficial for the function of AOP in the enhancement of the photocatalytic degradation of organic pollutants in an aqueous solution because the charge carriers can be generated more effectively after their bandgaps narrow. For instance, Li et al. constructed a series of hybrid Ag/TiO_2_ nanocomposites with different initial mass ratios by pulsed laser unfocused irradiation of the mixture of Ag/TiO_2_ nanomaterials in ethanol solvent (532 nm, 240 mJ/pulse, 8 ns) [46]. As shown in Figure 4, for the degradation of the methylene blue (MB) aqueous solution under a stable 250 W ultraviolet–visible lamp illumination (peak wavelength: 365 nm, range: 200–600 nm), the photocatalytic activities for pulsed laser-fabricated Ag/TiO_2_ nanocomposites are gradually optimized. Where the sample with an initial mass ratio of 1:4 displays superiority, a higher reaction rate constant of 0.152 min^−1^ and 100% MB removal rate can be achieved within 30 min, which is an enhancement rate 29.7 and 2.53 times higher than that of pure Ag (0.00512 min^−1^) and pure TiO_2_ (0.06 min^−1^), respectively. The enhanced mechanism is mainly attributed to the selective laser heating that induced the deposition of the Ag component on the surface of the TiO_2_ nanomaterials; the charge carriers from Ag and TiO_2_ can be resonantly excited and migrated to their surface to compound with the water species so that abundant ROS, such as the hydroxyl radicals with oxidation abilities, can be produced according to the AOP route.

Gondal et al. synthesized a series of nanocomposites of tungsten oxide and cadmium sulfide (WO_3_-*n*CdS, *n* = 0%, 5%, 15%, 30%, and 100%) by pulsed laser ablation in liquid (PLAL) [47]. The CdS nanospheres with different mass ratios are anchored on the surface of WO_3_ nanosheets by the pulsed irradiation of their mixture in deionized water (532 nm, 350 mJ/pulse, 5 ns). For the degradation of MB dyes under visible light illumination (λ > 420 nm), the WO_3_-30% CdS nanocomposite shows the highest photocatalytic activity, where the MB can be completely removed within 230 min; the corresponding reaction rate constant is 0.02 min^−1^, which is about 5.9-fold faster than that of pure WO_3_ (0.0034 min^−1^). The enhanced mechanism originated from the addition of CdS to WO_3_, reducing its bandgap energy, enhancing the visible light absorption, and reducing the electron–hole recombination through the Z-scheme charge transfer between WO_3_ and CdS. In addition, the same group also prepared a similar nanocomposite of TiO_2_-nCdS (n = 10%, 20%, and 40%) by PLAL for photodegradation of the persistent benchmark organic pollutant of methyl orange (MO) dyes in aqueous solution [48]. Here, as a narrow bandgap semiconductor, CdS is utilized to modify the wide bandgap of TiO_2_ at different percentages. Under the pulsed laser irradiation (532 nm, 350 mJ/pulse, 8 ns), a series of TiO_2_/CdS nanocomposites are obtained well, and the sample with a 10% percentage weight of CdS shows the best performance among all the samples. Ninety-seven percent MO can be removed within 60 min under the illumination of a 500 W xenon lamp, and the corresponding reaction rate constant is 0.0587 min^−1^. Compared with the same amount of pure TiO_2_, the photocatalytic activity is improved 1.45-fold. During the lamp irradiation, recombination of the electron–hole pairs in TiO_2_ can be controlled by trapping the electrons in CdS; so, the charge carriers can be effectively separated, and the best photocatalytic activities can be displayed. 

Choi et al. fabricated a series of plasmonic ZnO/Au@/Pd5 (@ = 5, 10, 15) nanocomposites by pulsed laser unfocused irradiation (532 nm, 40 mJ/pulse, 10 ns) of ZnO nanoparticles in Au-containing methanolic solution with different initial concentrations of 5, 10, and 15 wt. %, respectively; then, the Pd content (5 wt. %) was decorated on the surface of ZnO/Au nanospheres via an assisted chemical reaction [49]. For the degradation of MB dyes under a 200 W xenon lamp illumination (light density: 25 mW/cm^2^, with 420 nm UV cutoff filter), the ZnO/Au10/Pd5 shows superiority; 97% of the MB can be removed within 180 min, and the reaction rate constant is as high as 0.0145 min^−1^, which is an enhancement of 5.4 times compared with that of pure ZnO (0.0027 min^−1^). Moreover, a possible enhanced mechanism is suggested by the characterization of these nanomaterials; this is attributed to the synergistic effect between ZnO nanostructures and the nanoparticles of the noble metals Au and Pd; the introduction of noble metals promotes the concentration of free electrons and the enrichment of oxygen contents; the superoxide radical species trapped by the ZnO/Au10/Pd5 nanocomposite plays a dominant role in MB degradation via the AOP route. 

**Figure 5:**
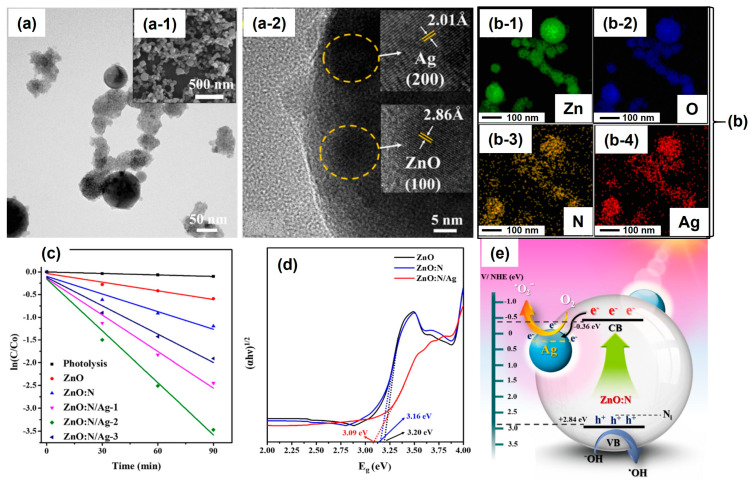
The morphological images, elemental components, and photocatalytic performances of pulsed laser-fabricated ZnO: N/Ag nanocomposites. (**a**) The morphological image of ZnO: N/Ag nanocomposite, (**a-1**) SEM, (**a-2**) HR-TEM. (**b**) Elemental mapping of the ZnO: N/Ag nanocomposite, (**b-1**) Zn, (**b-2**) O, (**b-3**) N, (**b-4**) Ag. (**c**) The dynamic fitting curve of photocatalytic degradation of rhodamine B (RhB) for different ZnO: N/Ag nanocomposites. (**d**) The estimated bandgap of ZnO, ZnO: N, and ZnO: N/Ag. (**e**) The schematic diagram of enhanced photocatalytic activity for ZnO: N/Ag nanocomposite. Reprinted with permission from Ref. [50].

As shown in Figure 5, the same group also prepared a silver nanoparticle-modified nitrogen-codoped zinc oxide nanocomposite (ZnO: N/Ag) by the pulsed laser ablation of a zinc target in a urea solution and a AgNO_3_ solution [50]. During the preparation of ZnO: N, a focused pulsed laser (Nd: YAG Surelite-Ⅱ, 1064 nm, 90 mJ/cm^2^) was utilized to ablate the Zn target immersed in a 10 mL urea solution for 30 min. Afterwards, the ablated nanocolloidal solution of Zn@ZnO: N was collected and further subjected to pulsed laser irradiation (Nd: YAG Surelite-Ⅱ, 532 nm, 100 mJ/cm^2^) for 30 min to achieve the good incorporation of N into Zn@ZnO. Additionally, the as-prepared ZnO: N solution was dispersed in the AgNO_3_ aqueous solution with different initial concentrations (denoted as ZnO: N/Ag-1, ZnO: N/Ag-2, and ZnO: N/Ag-3, respectively). These mixtures were also irradiated by using a Nd: YAG laser (Surelite-Ⅱ, 355 nm, 60 mJ/cm^2^) for 30 min to synthesize the ZnO: N/Ag nanocomposites. In Figure 5a, a morphological image of the laser-fabricated ZnO: N/Ag nanocomposite was illustrated. A spherical structure was mixed with other components (Figure 5(a-1)). To investigate this hybrid nanostructure, a high-magnification image was characterized under the high-resolution mode of TEM (see Figure 5(a-2)). The crystal lattices of Ag and ZnO can be observed; they correspond to the (100) plane of ZnO and (200) plane of Ag, respectively. For the specific analysis of the elemental components, the EDS mapping was utilized, and Zn, O, N, and Ag signals were synchronously detected (see Figure 5(b-1–b-4)). This confirms that the ZnO: N/Ag nanocomposite was successfully prepared.

In addition, for the photocatalytic degradation of rhodamine B (RhB) under a simulated sunlight irradiation (AM 1.5G, 350–1000 nm, 100 mW/cm^2^), the photocatalytic activities of ZnO: N/Ag were tested. As shown in Figure 5c, compared with pure ZnO and ZnO: N, the ZnO: N/Ag-2 exhibited the highest activity. A reaction rate constant of 0.038 min^−1^ was obtained; this was a 6-fold enhancement compared with that of pure ZnO (0.0063 min^−1^). After measuring the bandgaps of ZnO, ZnO: N, and ZnO: N/Ag, the enhanced mechanism was proposed. The bandgap of the ZnO: N/Ag nanocomposite was about 3.09 eV (redshift); this was narrower than those of pure ZnO (3.20 eV) and ZnO: N (3.16 eV), respectively (see Figure 5d). As shown in Figure 5e, narrowing the bandgap of the semiconductor was considered an efficient way to enhance the photocatalytic performance. This is because the electrons on the valance band (VB) of ZnO: N can be light-generated more effectively to increase the concentration of charge carriers on the conduction band (CB), and the holes on the VB can also be boosted to produce more superoxide radicals and hydroxyl radicals, respectively. 

Shipley et al., using PLAL, developed a series of laser-modified black TiO_2_ (marked as “LMB@-TiO_2_”, @ = 5, 15, 30, 60, 120). The anatase TiO_2_ nanopowders dispersed in deionized water were heated by laser irradiation at different times (laser parameters: 355 nm, 120 mJ/pulse, 3.6 ns), such as 5 min, 15 min, 30 min, 60 min, and 120 min, and their photocatalytic activities for MB degradation under visible light illumination (410–620 nm) were evaluated [51]. The morphologies and crystal structures of these samples were characterized using TEM and XRD measurements, respectively. Under the 355 nm laser ablation of the nanomaterials, a rutile shell was constructed on the surface of the pristine TiO_2_; this phase transformation between anatase and rutile TiO_2_ induced the formation of a heterojunction with a suitable photo-response to visible light. In particular, approximately 99% of the MB could be removed under the photo-chemical reaction of LMB120-TiO_2_ within 60 min. The superiority is attributed to the increasing density of active states in the region of anatase–rutile interfacial junctions, with higher charge transfers and accumulations; so the superoxide radicals and hydroxyl radicals can be generated to mineralize the MB molecules to H_2_O and CO_2_ according to the AOP mechanism.

Sánchez-Aké et al. fabricated a gold–palladium bimetallic alloy nanoparticle deposited zinc oxide film (Au/Pd/ZnO) by the pulsed laser-induced dewetting of nanoparticles [52]. In this process, an excimer KrF pulsed laser (COMPex Pro 201 from Coherent) with a 248 nm wavelength, 20 ns pulse duration, and 13.0 mJ average pulse energy was used to irradiate the samples with just one pulse. After fabrication, for photocatalytic degradation of indigo carmine (IC) dyes in aqueous solution (light source: solar simulator lamp, Oriel, 150 W, 360 W/m^2^), the sample of ZnOAu30Pd30 displayed the highest activity. Ninety-nine percent of the IC can be decreased within 300 min, with a higher rate constant of 5.5 × 10^−3^ min^−1^. This is about 6.1-fold higher than that of pure ZnO film. The enhanced mechanism was mainly attributed to the Schottky barrier (SB) constructed between the Au-Pd alloy and ZnO. Among a series of noble metals, the Au-Pd metals have larger work functions; so, the electrons can be transferred from the semiconductor of the n-type to the noble metals. Therefore, the light-generated electrons and holes in the energy bands of the semiconductors can be effectively separated to produce abundant ROS in the photocatalytic system [52]. 

**Figure 6:**
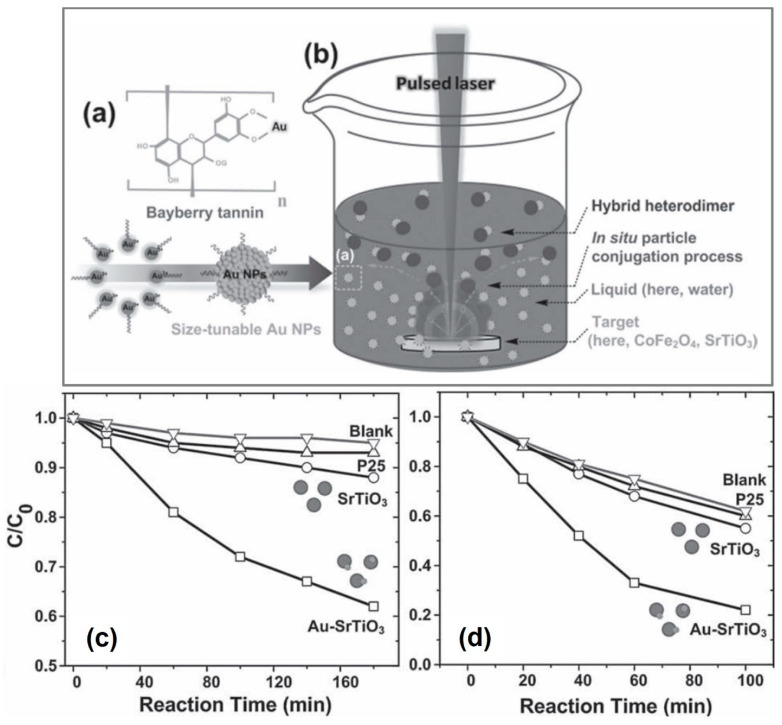
The schematic diagram of size-tunable Au nanoparticles (**a**). Schematic diagram of pulsed laser ablation of solid targets in liquids (**b**). The photocatalytic degradation of rhodamine B (RhB, (**c**)) and methyl orange (MO, (**d**)) dyes in a water system under the commercial TiO_2_ (simply marked as “P25”), pure SrTiO_3_, and the laser-fabricated Au-SrTiO_3_ nanocomposites. Reprinted with permission from Ref. [53].

Chu et al. prepared a series of hybrid Au-SrTiO_3_ and Au-CoFe_2_O_4_ nanocomposites by PLAL for the photocatalytic degradation of rhodamine B (RhB) and MO under visible-light irradiation (λ > 420 nm) [53]. As shown in Figure 6a,b, a 248 nm KrF excimer laser with an energy density of 1.2 J/cm^2^ was utilized to ablate the SrTiO_3_ and CoFe_2_O_4_ ceramic targets, and the laser-generated species were composited with the size-tunable Au particles (size: 2.5~6.5 nm) from the HAuCl_4_ precursors under the controlling of the concentration of bayberry tannin (BT). In Figure 6c,d, in comparison with the pure SrTiO_3_ and commercial TiO_2_ (marked by “P25 aerosol”), the Au nanoparticle-modified SrTiO_3_ displays a more active degradation ability for both RhB and MO; the reaction rate constants from their dynamic degradation curves are synchronously higher, respectively. The enhanced mechanism is ascribed to the formation of Schottky junctions in the Au and SrTiO_3_ interfaces; for SrTiO_3_, the recombination of electron–hole pairs can be effectively inhibited by the coupling effect of relative energy bands, and the electrons from Au nanoparticles can also be trapped by the valance states of Ti^3+^ ions via a Coulombic action to produce more superoxide radicals surrounding the surface of SrTiO_3_. Moreover, for Au particles with small sizes of 2.5~6.5 nm, the SPR effect can be sufficiently utilized under the excitation of visible light irradiation; so, the visible light can be absorbed more effectively to boost the separation of electron–hole pairs.

### 3.3. Catalytic Reduction of 4-Nitropenol and Nitrobenzene in the Water System

Apart from the organic dyes in water systems, such as MB, MO, IC, and RhB, there are some other organic pollutants dispersed in water that are also difficult to remove, and catalytic technology is utilized to degrade these hazardous materials. In particular, the toxic nitrophenol and nitrobenzene are widely used in petrochemical synthesis, including paints, pulp, plastics, rubber, pesticides, and dye production [54], but the wasted products are also harmful to the environment. Therefore, the 4-nitrophenol (4-NP) and nitrobenzene that exist in industrial wastewater have aroused great concern due to the discharging of nitrophenol seriously affecting the health of nearby human beings; it has been listed as a priority pollutant by the U.S. Environmental Protection Agency (US. EPA). Nowadays, their removal by catalytic reduction is considered a promising approach; not only can the reduction of toxic 4-NP be achieved but neat products can also be obtained. The key point to realize in this conversion is the development of a series of catalysts with high performances, and now, the pulsed laser technology has also been utilized to prepare these catalysts.

**Figure 7:**
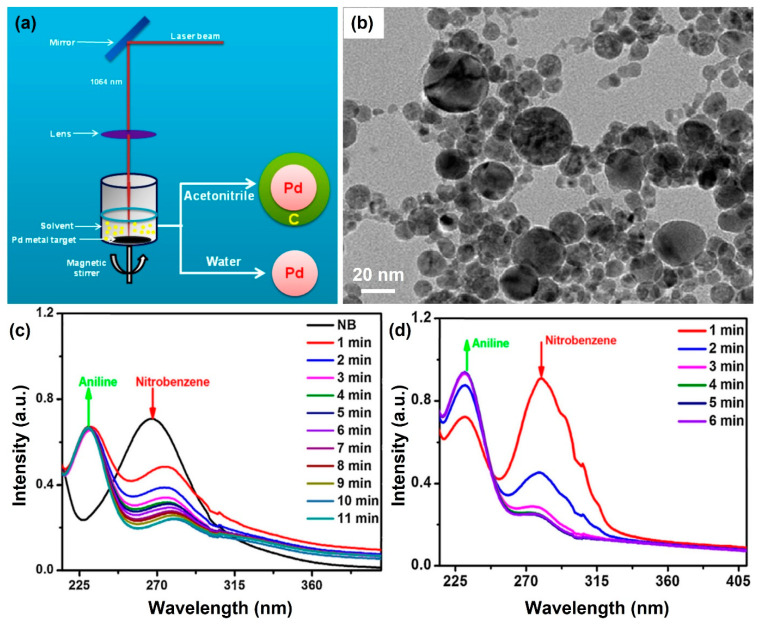
Synthesis of core–shell Pd@C and Pd nanospheres by using pulsed laser-focused ablation in acetonitrile and water, respectively (**a**). The morphological image of the synthesized Pd nanospheres (**b**). The UV–Vis absorption spectra for catalytic reduction of nitrophenol to aniline with Pd nanospheres (**c**). The UV–Vis absorption spectra for catalytic reduction of nitrophenol to aniline with Pd@C nanocatalysts (**d**). Reprinted with permission from Ref. [55].

For instance, Kim et al. synthesized a graphitized carbon-encapsulated palladium (Pd) core–shell (marked as “Pd@C”) nanosphere for the catalytic reduction of nitrobenzene to aniline by the pulsed laser ablation of a solid Pd target in liquids [55]. As illustrated in Figure 7, the Pd metal targets dispersed in the liquids (acetonitrile or water) can be ablated as a form of plasma species to become the single-crystalline cubic metallic Pd spheres (Figure 7a). Meanwhile, the acetonitrile, after high-energy laser-focused ablation, can be graphitized (1064 nm, 8.42 J/cm^2^, 10 Hz), and it served as a shell layer on the surface of the Pd core (Figure 7b). Compared with the pure Pd spheres, the laser-synthesized core–shell Pd@C nanospheres exhibited a higher catalytic performance for the catalytic reduction of nitrobenzene in the presence of sodium borohydride (NaBH_4_). For Pd@C, a reaction rate constant of 0.24 min^−1^ can be calculated from the pseudo-first-order reaction kinetic model (i.e., −ln(C/C_0_) = *kt*; *k* is the reaction rate constant), which is about 3.03 times higher than that of pure Pd spheres (0.079 min^−1^). The corresponding time-dependent UV–Vis absorption spectra are illustrated in Figure 7c and Figure 7d, respectively. The enhanced mechanism originated from the stacking interaction of graphitized carbon layers adhered to the surface of Pd catalysts. The aggregation of Pd nanospheres can be prevented by carbon layers, which boost the adsorption rate of nitrobenzene onto this catalyst because the surface area can be increased to provide efficient adsorption and transport of charge carriers. The electron concentrations can be also increased owing to an efficient interfacial electron transfer from the graphitized carbon layers to the Pd nanostructures, which in turn leads to an efficient transfer of electrons from the BH_4_^−^ (donor) to nitrobenzene (acceptor) through the Pd catalyst, resulting in a highly efficient reduction activity.

**Figure 8:**
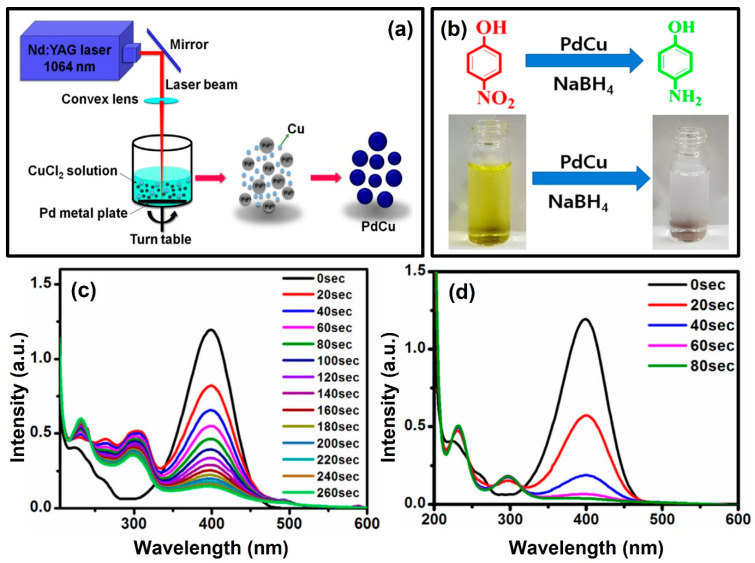
The schematic diagram for preparation of PdCu nanostructures by pulsed laser ablation of Pd plate in liquids assisted with a chemical approach (**a**). Optical photograph of catalytic reduction of 4-nitrophenol to 4-aminophenol with PdCu nanocatalysts (**b**). The UV–Vis spectra for the reduction of 4-nitrophenol to 4-aminophenol with Pd nanocatalysts (**c**). The UV–Vis spectra for the reduction of 4-nitrophenol to 4-aminophenol with PdCu-1 nanocatalysts (**d**). Reprinted with permission from Ref. [56].

As shown in Figure 8, the same group also prepared a series of hybrid PdCu nanostructures (marked by “PdCu-0.5”, “PdCu-1”, “PdCu-2”, and “PdCu-3”, respectively) by using pulsed laser-focused ablation in liquids and the assistance of a chemical route (see Figure 8a) [56]. Specifically, the Pd metal plate dispersed in deionized water was ablated by a high-density pulsed laser ablation (the parameters were also set as 1064 nm, 8.42 J/cm^2^, 10 Hz); the ablated plasma species were quenched in deionized water and then synchronously reacted with the CuCl_2_ aqueous solutions under different concentrations (0.5, 1, 2, and 3mM). In the excess of NaBH_4_, for the catalytic reduction of 4-nitrophenol to 4-aminophenol, the catalytic activities of PdCu catalysts were evaluated (see Figure 8b). Therefore, compared with the pure monometallic Pd, the PdCu-1 catalyst displays the highest performance and stability; a 99.47% conversion rate and a 0.04495 s^−1^ reaction rate constant can be obtained (see Figure 8d; 4-nitrophenolate can be reduced to 4-aminophenol within 80 s), which is about 5.91 times higher than that of pure monometallic Pd nanostructures (Figure 8c). To analyze the related enhanced mechanism, the turnover frequency (TOF) parameters of the bare Pd and PdCu-1 bimetallic nanocatalysts were estimated to be 1.31 × 10^−6^ and 4.25 × 10^−6^, respectively. This confirms that the PdCu-1 bimetallic nanocatalysts are more active than bare Pd nanostructures. 

Choi et al. fabricated a graphitic carbon-encapsulated Au nanoparticle (marked by “Au@GC”) by pulsed laser ablation in a methanol, hexane, and acetonitrile solvent [57]. During the interaction between the laser and the Au plate in liquids, the Au nanoparticles can be easily ablated from the target Au plate, and the carbon layers can also be obtained under a proper laser-focused ablation in these carbon-containing solvents (1064 nm, 80 mJ/pulse, 10 Hz). For the reduction of 4-nitrophenol, the catalytic activities of composited Au@GC and Au nanoparticles were evaluated in the NaBH_4_ environment. Here, in contrast to the similar studies above, the Au@GC fabricated in methanol displayed a lower catalytic performance than that of the pure Au nanoparticles synthesized in deionized water. For Au@GC and pure Au, respectively, 76% and 85% 4-nitrophenol could be decomposed within 3 min; the corresponding reaction rate constants were calculated as 8.30 × 10^−3^ s^−1^ and 11.9 × 10^−3^ s^−1^. According to the TEM, Raman, and XPS analysis, the mechanism can be attributed to the strong binding of 4-nitrophenol at the catalytically active sites of Au nanoparticles; due to the large formation energy of their higher surface areas, the 4-nitrophenol can be effectively adsorbed and decomposed. Meanwhile, for the Au nanoparticles encapsulated by graphitic carbon layers, the active sites are blocked by the partial and imperfect carbons; so, the catalytic activity is concealed.

Mostafa et al. prepared a kind of core–shell Au@ZnO nanocatalyst by pulsed laser-focused ablation of pure granulated zinc metal in chloroauric acid (HAuCl_4_·3H_2_O, laser paramaters:1064 nm, 60 mJ/pulse, 10 Hz) [58]. For the catalytic degradation of 4-nitrophenol in the presence of NaBH_4_, the catalytic activities of pure ZnO and Au@ZnO nanocatalysts were evaluated, respectively. For the pure ZnO nanoparticles, 90% of the 4-nitrophenol could be degraded within 12 min, with a reaction rate constant of 0.2283 min^−1^. For the Au@ZnO nanostructure, only 4 min was needed to achieve the same degree of conversion, and the corresponding reaction rate constant was as high as 0.751 min^−1^, which was about 3.29 times higher than that of the pure ZnO nanoparticles. In particular, just like photocatalysis under light excitation, during these catalytic procedures the presence of NaBH_4_ is significant. In Au@ZnO nanostructures, the electrons can be transferred across the interface of hybrid Au and ZnO; the fast electron transfer in turn can increase the local electron concentration and facilitate effective electron transfer from BH_4_^-^ to 4-nitrophenol. Here, as a crucial bridge of electron transfer, the BH_4_^-^ ions dispersed in aqueous solution can be reacted with the surface of Au@ZnO nanostructures and produce a surface hydrogen species on the surface of Au@ZnO nanoparticles. With the help of the surface hydrogen species, the 4-nitrophenol can be reduced to the nitroso compound and then further reduced to hydroxylamine and aminophenol.

In addition, apart from the catalytic reduction of organic pollutants, laser-prepared nanocomposites are also utilized to kill pathogens in the water environment. For example, Li et al. fabricated a hybrid Ag/CeO_2_ nanocomposite by selective laser welding in a liquid [42]. The synthesis principle was similar to the case illustrated in Figure 1. During the preparation, the Ag nanoparticles can be melted and welded with the CeO_2_ nanosheets under a 532 nm laser output. With the catalytic antibacterial of Staphylococcus aureus (*S. aureus*) under visible light illumination (λ > 420 nm, 150 W), the laser-synthesized nanocomposite exhibited a higher performance, and an 82.4% sterilization rate was achieved, which was 2.93 and 2.99 times higher than those of pure Ag and pure CeO_2_, respectively, according to the component analysis with X-ray photoelectron spectroscopy (XPS), X-ray diffraction (XRD), and the theoretical structures. The enhanced mechanism was attributed to the coupling of visible light harvesting and the enrichment of oxygen species on the surface of CeO_2_; the light-generated charge carriers could be reacted with the oxygen species more effectively. Hence, the ROS can be produced, and the *S. aureus* can be killed via a special superoxide dismutase (SOD) route. Therefore, with the development of laser technologies, more and more regions will benefit from this powerful tool. 

### 3.4. Discussion on Laser Technology for Pollutant Removal in the Water System

In previous reports, the progress on pulsed laser-prepared nanomaterials in liquids, such as various quantum dots, nanospheres, nanosheets, and nanocomposites, are summarized, respectively, and the specific mechanisms are also introduced in detail [59,60,61]. In recent years, this technology has been applied in many significant fields, such as environmental remediation, biomedical science, and perovskite solar cells [62,63,64]. Here, focusing on wastewater remediation, some innovative works and advantages of pulsed laser-made nanocatalysts for catalytic removal of organic pollutants in water systems are critically pointed out. The catalytic activities of laser-prepared nanomaterials are generally enhanced in the investigations of laboratories. However, to meet the practical requirements of specific industries, the yields of laser-fabricated nanomaterials need to be increased (they are at about mg level at present), and thus, the potential of this technology for massive applications can be sufficiently displayed. The weakness of yield production is mainly restricted by the handmade precursor samples; the samples dispersed in a little bottle can only be heated step by step with the assistance of a manual and a proper laser source output. At present, if the sample-supported platform can be replaced by an automatic pattern, the yields of nanomaterials can be effectively improved. 

**Figure 9:**
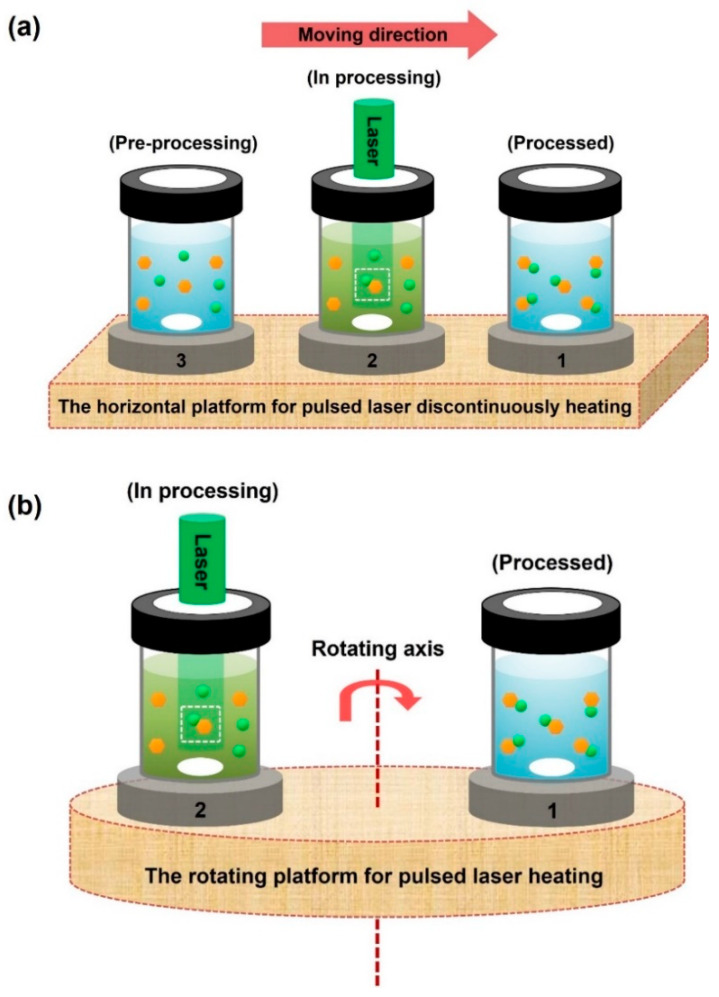
The schematic diagram of the automatic platform for sample heating by a stable laser. (**a**) The horizontal platform. (**b**) The rotating platform.

In this review, assuming that the heating laser source is stable, two types of automatic platforms are theoretically proposed. As shown in Figure 9, to realize the automatic heating of the samples, the horizontal and rotating platforms are schematically designed, respectively. In Figure 9a, after the first sample processing (see sample 1, the sample has been processed by laser source), the next one can be processed with the platform moving in a line direction (see sample 2, the sample is in processing, and sample 3 is waiting for processing). In Figure 9b, the shifting of samples is controlled by a rotating axis, and the samples can be processed in a circular line (see samples 1 and 2). If the working space of the platform is big enough, the yields of the production can be naturally expanded and even improved many times in comparison with that of a single bottle. Generally, the automatic control of the platform can be theoretically realized by special software, which can be achieved with the technical assistance of a software engineer. Additionally, with the development of high-power lasers and by taking these lasers as heating sources, the volume of samples also can be expanded to produce large amounts of products. 

Here, the laser technology is focused on fabricating nanocatalysts for catalytic removal of organic pollutants in water systems. In addition, as a topic of discussion, laser technology also can be utilized to detect the components in inorganic materials, including the elemental contents and molecular radicals inside [65,66]. This detecting technology is well known as laser-induced breakdown spectroscopy (LIBS), which is a popular way to realize the rapid detection of contents. The general principle is that the components on the surfaces of the samples can be ablated and excited by the high-density laser beams into becoming plasma, and the elemental signals of excited components can be detected by using a grating spectrometer [67]. According to the detected signals, the elemental information can be recognized by comparing it with the standard signal lines. In recent years, our group also reported a series of studies in this field [68,69,70], including solid, liquid, and gas phases. Moreover, if the water system is affected by microorganisms, such as Gram bacteria and viruses, these pathogens can also be killed by the high-density laser irradiation in water, and the water quality can be treated well. This is because their protein insides can be inactivated by the powerful laser heating effect. The temperature caused by laser interaction is significantly higher; in the same way, the noble metals can be melted by the proper laser sources.

## 4. Summary and Outlook

In summary, this review briefly summarized the recent progress of laser-made nanomaterials for the catalytic removal of organic pollutants from water systems, such as organic dyes, nitrophenol, and nitrobenzene. To achieve an excellent catalytic performance, the method to fabricate nanocatalysts with a neat surface, high crystallinity, and low cost is crucial. As an innovative approach for nanocatalyst preparation, pulsed laser heating in liquids demonstrates rapid heating and liquid quenching; the nanomaterials after laser treatment can be reshaped and modified with other components under the critical control of initial parameters (laser, precursors, or liquids). Hence, for the catalytic degradation of organic pollutants in water systems, with the design of a nanostructure by laser heating (irradiation or focused ablation) in liquids for the generation of reactive species the catalytic activities can be precisely optimized. In particular, for the photocatalytic degradation of organic dyes in water systems, the bandgaps of photocatalysts should be narrowed to boost visible light harvesting, such as by the coupling of special components (noble metals, sulfide, and polyoxometalates). For the catalytic reduction of nitrophenol in water, the presence of NaBH_4_ is necessary; as a medium for electron donors, the charge carriers can be effectively transferred to produce the surface hydrogen species. Moreover, even though the catalytic performances of laser-made nanomaterials at present are excellent, with regard to industrial applications the production yields still need to be improved because the handmade samples are rather limited. For massive applications, automatic technology and high-power lasers should be introduced to improve the yields of production. In this review, two strategies of automatic platforms, the horizontal and rotating platforms, are theoretically proposed. In the future, a series of samples can be continuously fabricated by these instruments. Meanwhile, with the development of high-power lasers, the volume of samples can also be naturally expanded. Additionally, laser technologies for other applications, such as elemental detection and the killing of pathogens, are also briefly discussed. Overall, this review provides a valuable reference for the cross-investigation of laser technology in liquids and nanomaterials in environmental catalysis, giving it great potential for the development of advanced catalysts.

## Figures and Tables

**Table 1:** The enhanced photocatalytic activities of different nanocomposites.

Nanocomposite	Synthesis Method	Photocatalytic System	Performance	Reference
ZrS_4_-Znln_2_S_4_	Hydrothermal	Tetracycline	3-fold enhancement	[18]
Ag/ZnO	Pulsed laser melting	Methylene blue dyes	2-fold enhancement	[19]
graphene/CoO@Co	Impregnation–calcination	Chlortetracycline hydrochloride	100% removal ratio within 12 min	[20]
TiO_2_/BP/g-C_3_N_4_	Pulsed laser ablation	Water splitting	5.4-fold enhancement	[21]
WO_3_/BP/g-C_3_N_4_	Pulsed laser ablation	Water splitting	5.3-fold enhancement	[22]
NLDH-NOG	Chemical approach	Hydrogen productionSA degradation	4.5-fold enhancement1.9-fold enhancement	[23]
DBS-ZnCuCo LDH	Hydrothermal method	Hydrogen production	1.5-fold enhancement	[24]
NM-WO_3−x_@MC	self-assembly	Hydrogen production	5.2-fold enhancement	[25]

**Table 2:** The enhanced photocatalytic activities for organic dye degradation under different nanocomposites.

Nanocomposite	Degradation System	Light Source	Performance	Reference
Ag/TiO_2_	MB solution	λ ~ 200–600 nm	2.53-fold enhancement	[46]
WO_3_-nCdS	MB dyes	λ > 420 nm	5.9-fold enhancement	[47]
TiO_2_-nCdS	MO dyes	500 W xenon lamp	1.45-fold enhancement	[48]
ZnO/Au@/Pd5	MB dyes	200 W xenon lamp	5.4-fold enhancement	[49]
ZnO: N/Ag	RhB dyes	350–1000 nm	6-fold enhancement	[50]
LMB@-TiO_2_	MB dyes	λ ~ 410–620 nm	99% removal rate in 60 min	[51]
Au/Pd/ZnO	IC dyes	150 W solar lamp	6.1-fold enhancement	[52]
Au-SrTiO_3_	RhB/MO dyes	λ > 420 nm	With superiority	[53]

## Data Availability

Not applicable.
